# Therapists’ problematic experiences when working with obsessive-compulsive disorder: a qualitative investigation of schema modes, mode cycles, and strategies to return to healthy adult mode

**DOI:** 10.3389/fpsyt.2023.1157553

**Published:** 2023-12-15

**Authors:** Suzana Semeniuc, Maria Cristina Sterie, Camelia Soponaru, Simona Butnaru, Ovidiu Gavrilovici

**Affiliations:** ^1^Faculty of Psychology and Education Sciences, Alexandru Ioan Cuza University, Iasi, Romania; ^2^“Constantin Rădulescu-Motru” Institute of Philosophy and Psychology, Department of Psychology, Romanian Academy, Bucharest, Romania

**Keywords:** obsessive-compulsive disorder, therapeutic relationship, schema therapy, self-refection, healthy adult

## Abstract

**Introduction:**

Obsessive-compulsive disorder (OCD) is one of the most challenging pathologies for therapists, being chronic and often characterized by frequent relapses. The therapeutic relationship plays a critical role in the outcome of therapy. The dynamic interaction between therapist and client schema modes determines the quality of the relationship.

**The objective:**

of the present qualitative research is to investigate and conceptualize the triggers for therapists when working with OCD clients, the therapists’ schema modes that are activated, and the strategies they use to get back into the Healthy Adult mode.

**Method:**

Using the in-depth interview technique, we interviewed 15 psychotherapists of various therapeutic orientations. After several demographic items, the therapists answered some introductory questions about their general perceptions of working with this pathology. They were then guided to go through a specific therapeutic situation in imagery that they identified as particularly difficult to manage in the therapeutic relationship. For data analysis, we used the interpretative phenomenological analysis (IPA) method and thematic analysis.

**Results:**

For therapists working with clients with OCD, two main categories of triggers have been identified: Perceived resistance to change and Superiority. Two other categories, Client immobilized by conflict and Abusive, emerged from our analysis. Therapists’ reactions to triggers were synthesized into mode processes that (1) were not acted on and (2) were displayed in relation with the client. For mode processes that therapists did not explicitly act on, there was triggering of the Vulnerable Child in relationship with a Demanding Parent, followed by various coping modes, depending on the category of trigger, and in the displayed mode, most therapists operated in the Healthy Adult mode. With respect to the process, strategies used by therapists to return to Healthy Adult mode that stood out were: focusing on the process, self-acceptance, self-compassion, and somatic grounding strategies, and focusing on the client’s resources.

## 1 Introduction

Obsessive-compulsive disorder (OCD) is characterized by the presence of obsessions–thoughts, impulses, or images that are repetitive, intrusive, ego-dystonic, and cause significant emotional distress. The person attempts to avoid this distress through suppression or neutralization mechanisms, using various types of mental or behavioral compulsions. The whole process significantly reduces the individual’s functionality (1). OCD is usually chronic–two-thirds of cases become chronic (2)–and begins between the ages of 11 and 28. The disorder is often complicated by multiple comorbidities and is reported to affect between 1.2 and 3% of the population in studies from different countries (3). Currently, cognitive-behavioral psychotherapy (CBT) and psychopharmacological treatment are two types of therapeutic interventions that have been validated as having a significant effect on reducing symptoms. Regarding CBT, only 50–60% of those affected complete the entire therapeutic protocol, 25% refuse the Exposure with Response Prevention technique–the key element of the intervention–and a quarter do not achieve significant benefit from treatment (2). Overall, none of the available interventions provide a significant therapeutic response in approximately 30% of people with OCD (3). In clinical practice, relapses are also common in those who initially respond to some form of treatment.

Developed by Jeffrey Young in the 1990s to address CBT-resistant disorders (4), particularly personality disorders, schema therapy (ST), which is essentially a trans-diagnostic approach (5), has expanded rapidly in recent years to provide explanatory models and intervention protocols for OCD, among other disorders. The theory underlying ST operates with two main concepts: early maladaptive schemas and schema modes. The frustration of basic emotional needs in childhood and adolescence leads to the formation of what are known as early maladaptive schemas (EMS). These are pervasive patterns of information organization consisting of memories, feelings, emotions, and cognitions that influence perceptions of the self, others, and relationships (4) and play a critical role in the risk of developing certain pathologies in adulthood. When the person encounters situations that are more or less similar to those that led to the original development, the schema is activated. The person experiences intense negative affects to which he or she must adapt. The coping modalities to which the person resorts are divided into three general categories, based on the three automatic responses to danger, that is, fight, flight, or freeze: overcompensation (the person behaves contrary to the schema), avoidance (the person adopts various detachment behaviors or avoids situations that may activate the schema) and surrender (the person considers the schema to be true and behaves in accordance with it) (4, 5). The concept of mode refers to a mental state resulting from schema activation that includes specific cognitive, emotional, and behavioral aspects when a schema represents a stable feature in the personality structure (4). A person may move rapidly from one mode to another or may persist in the same mode for longer periods of time (5). In ST, the modes are divided into the following general categories: dysfunctional child modes (expressing needs and emotions, characterized by intense negative affect–vulnerable child, angry child, impulsive child, etc.), dysfunctional parent modes (parts representing introjected dysfunctional parental responses–critical, punitive, demanding parent), healthy modes (happy child and healthy adult) and dysfunctional coping modes (surrender, avoidant, or overcompensating coping modes) (4–6). With regard to EMSs, recent studies have not shown specificity for people with OCD, and the heterogeneity characteristic of obsessive spectrum disorders is also found in their early maladaptive schemas (7). Research investigating EMSs associated with OCD or compared with other disorders or the general population reports significantly higher scores across a variety of schemas: Dependency/Incompetency, Vulnerability to Harm and Illness, Abandonment/Instability, Lack of Self-control/Self-discipline, Social Isolation, Emotional Deprivation, Enmeshment/Undeveloped self, Entitlement, Subjugation, Approval-seeking, Negativity/Pessimism, Unrelenting standards, Defectiveness/Shame, and Failure (8–18). Vulnerable Child, Angry Child, Punitive Parent, Demanding Parent, Perfectionist Overcontroller, Self-aggrandizer, and avoidant modes in the form of Detached Protector and Detached Self-Soother have been associated with OCD and obsessive personality type (5, 6, 18–21). Parenting styles characterized by the intolerance of mistakes, excessive emphasis on moral principles, achievement, unrealistic standards, and inflicting punishment by criticizing, excessive blaming, or rejection seem to be a consequence of early maladaptive schema patterns in OCD sufferers (22–24). ST interventions for individuals with OCD, either on their own or integrated into CBT protocols, have been shown to be effective in decreasing symptoms (25–28). One of the main components of ST interventions is the therapeutic relationship, which is based on the concept of “limited reparenting” (4, 5). Although not all therapeutic guidelines consider the client–therapist relationship as an intervention tool in itself, as is the case in ST, there is a general consensus that any therapy involves a human interaction that can positively or negatively affect the outcome of the intervention (29). For people suffering from OCD, the therapeutic relationship can be a determining factor in treatment success, as it combines empathic support with empathic confrontation to facilitate change (4, 5, 30). Most previous studies of the therapeutic relationship with people with OCD are quantitative, based on the client’s perspective, and have essentially focused on measuring its effects on treatment outcomes (31–35). Considerably fewer studies have focused on exploring therapists’ experiences of the therapeutic relationship with people with OCD (36). Studies that have explored the therapeutic relationship from the perspective of ST in general, without reference to a specific pathology, provide support for the idea that the dynamics of the client–therapist relationship may be profoundly influenced by the maladaptive schemas of the two as well as the dynamics of the schema modes they activate (37–39). The risk, in the absence of awareness of these issues, is that the therapeutic relationship, rather than being a ‘corrective experience’ (4, 5) for the client, constitutes a new experience that confirms and reinforces pre-existing maladaptive schemas. We therefore believe that the present qualitative study provides valuable insights into the dynamics of the therapeutic relationship from the perspective of therapists of various orientations who treat people suffering from OCD.

## 2 Materials and methods

### 2.1 Objectives

The main objective of the present study was to explore the therapists’ perspective, from both a rational and emotional approach, on the client–therapist dynamics that are difficult to manage in the therapeutic relationship when working with OCD pathology. Thus, we aimed to identify triggers when working with OCD clients, therapists’ schema modes activated by these clients, and what strategies, if any, therapists use to activate their Healthy Adult mode.

### 2.2 Participants

Participants were recruited through invitations sent to psychotherapy associations that are active in Iasi, Romania. Inclusion criteria were (1) practicing psychologists trained in a recognized school of psychotherapy in Europe who (2) were treating clients diagnosed with OCD. Nineteen psychotherapists responded and agreed to be interviewed while maintaining their anonymity. Two of these were excluded because they had not treated clients diagnosed with OCD. Two imageries were removed from the analysis because they dealt with difficult to manage situations involving other people (a family member and the attending physician) who were not directly involved in the therapeutic process. Recruitment began on 15 September 2022 and closed on 30 November 2022. The final sample comprised 15 participants (13 women). The average age of participants was 38.9 years and the average years of experience in the profession was 9.13 years. Most of the participants had basic training in Ericksonian psychotherapy (6), systemic therapy (5), and CBT (6), with a proportion (6) having training in two or more schools of therapy. Two of the participants had additional training in ST. For a detailed demographic description of the group of participants, see Table 1. All participants in the study work mainly with adults (only one therapist also works with adolescents) with various pathologies, some having more or less experience with OCD. In the population in which the study was conducted, i.e., in Romania, there is no formal specialization to work with a specific pathology. Most therapists work with patients with different pathologies. Of the situations depicted in the imagery, only one was followed by the client dropping out. In the remaining cases, therapy continued after the situation described.

**Table 1:** Demographic data of the participants.

Sex		Age	Experience Training	
		(Years)	(Years)	
	f	32	7	Systemic
	f	25	1	Integrative
	f	49	13	CBT
	f	44	18	CBT, ST
	m	36	12	Ericksonian psychotherapy
	f	49	12	Ericksonian psychotherapy
	f	35	4	Systemic
	f	26	4	Systemic
	f	38	10	Systemic
	f	30	4	Systemic, CBT
	f	42	7	Psychodrama, ST, CBT
	f	35	7	Ericksonian Psychotherapy, ST
	f	59	15	Ericksonian Psychotherapy, Narrative Therapy
	m	40	11	Ericksonian Psychotherapy, CBT
	f	44	12	Ericksonian psychotherapy

### 2.3 Research team

The first two authors are certified in ST and the second author is a certified trainer in ST. All five authors are psychologists, four of whom have clinical work and training in several schools of psychotherapy (CBT, Narrative Therapy, Ericksonian Psychotherapy, and Experiential Therapy), as well as having conducted academic and research work.

### 2.4 Procedure

In our study, we used the in-depth interview technique, in online format, using the Zoom application. All participants were assured of confidentiality by the research team and gave their consent for the interviews to be video recorded. The researchers used a structured approach to interviewing (40). They developed an interview guide that included some demographic questions and a question about general perceptions of working with OCD pathology designed to create a safe and comfortable environment for participants. Then participants were asked to go through a structured imagery exercise, based on the diagnostic imagery technique (41, 42). Each therapist was instructed to choose a situation from a therapy session with an OCD client that they felt at the time was the most emotionally difficult to manage from the therapy sessions they conducted with this type of pathology. Participants were asked to close their eyes or look down, bring the situation to mind and describe, sequence by sequence, in the present tense, what was happening in the image. The script that was used by the researcher to conduct all of the imagery sessions contained the same questions in all of the sequences: “What is going through your mind at this moment? What emotions are you experiencing? What sensations do you notice in your body? What happens next?” No suggestions were offered either with respect to how therapists were working to return to Healthy Adult mode or with respect to their experience of their own needs in that moment. Imagery sessions lasted between 15 and 40 min. The imagery ended when it reached a natural conclusion. Full interviews, including debriefing, lasted on average 60 min. We did not have a pre-set word count or minimum length as criteria. For inclusion in the analysis, the imagery process needed to include significant emotional activation of the therapist. No imagery was excluded on this criteria. The last section of the interview involved work with debriefing the therapist.

### 2.5 Data analysis

Following an approach based on the interpretative phenomenological analysis (IPA) method (43), each imagery was analyzed as a whole. In the first stage, we transcribed the data from the video recordings. The first two authors independently carried out the analysis by watching the transcripts and video recordings in parallel. Thus, the following general questions regarding the therapist, the client, and the interaction were answered as we examined each recording. Regarding the therapist, we considered whether the therapist seemed triggered, which child and/or coping modes were activated, whether the therapist was at least partially aware of them, and if so, what strategies the therapist used and to what extent the therapist succeeded in acting from Healthy Adult mode. Regarding the client, we observed in each sequence described what the presenting problem was, which schema modes seemed to be activated, whether the client remained in the same modes, fell into other dysfunctional modes, or activated Healthy Adult mode following therapist interventions. In terms of the interaction, we examined, in each sequence described, what types of dysfunctional mode cycles were occurring, whether the therapist was aware of them, and whether the therapist was effective in interrupting these cycles. We also carried out a thematic analysis in terms of the identification of schema modes. As a codebook for the highlighted schema modes, Edwards’ (6) list and definitions of modes in schema therapy were used. For each example, the first two authors summarized their findings independently and then compared them, discussing the differences and agreeing on a common summary. The next step was to carry out a cross-case comparison, in which we looked for similarities and differences between all the cases. The other three members of the research team individually reviewed the summary of each case and completed a spreadsheet answering four questions: what was the trigger for the therapist in terms of schema modes, what was the automatic response to the trigger in terms of schema modes, how was the response displayed in the relationship with the client, and what strategies did the therapist use to respond from the Healthy Adult mode. The responses were then compared by analyzing similarities and differences. In discussing differences between evaluators, the relevant sections of the video recordings were reviewed. For each case, a common consensus table was created with the responses to the four questions. We then organized the results into categories.

## 3 Results

The results has been grouped into three sections, described in detail below. The first section relates to the client behaviors that acted as triggers for the therapist. The second section relates to the therapist’s schema modes that were activated during the session with the client and relived in the imagery. The last section relates to the therapist’s strategies for accessing the Healthy Adult mode.

### 3.1 Triggers for therapists

Analysis of the imagery exercises revealed two main categories of triggers for therapists: clients perceived as resistant to change (Perceived resistance to change—divided in three subcategories: Angry resistance, Avoidant resistance, and Blended resistance) and clients perceived as having a demanding and superior attitude (Superiority). Two other categories (Client immobilized by conflict and Abusive) emerged from three particular situations encountered in the imagery exercises.

Most of the mentioned triggers belong to the category Perceived resistance to change. In the Angry resistance subcategory, we encountered the following examples:

(P 11) “*As he takes off his jacket and puts it on the rack, he already starts telling me: I do not believe in this method you gave me. I’m not sure this works.. If I believed it even a little bit, I’d probably try it. And that’s why I do not think this thing makes me feel better.*” The therapist perceives the rejection of any help by the client who we identified as being in Help-rejecting complainer mode.

(P 1) “*On the left is a lady who has the same ideas as the client with OCD. This lady* [another participant in the client’s therapy group] *is claiming that she is not addicted, that she can control her consumption. The client almost jumps out of his chair and points at her, saying see, see, is that right? I think so too. You’re right, that’s the truth.”* The therapist perceives that the client is supporting the other member who is claiming she is not addicted and felt attacked, like the two were ganging up against her, which suggests an Overcompensator mode (Self-aggrandizer, Scolding Overcontroller) with Defiant and Impulsive child mode backstage.

(P3) *“We are at her house, on the couch where we used to meet, in a living room, but in a certain part of the room, because we were not allowed to touch an armchair with a certain quilt that the husband sat on. She would not go into her bedroom and would not touch things in her bedroom because the husband, it seemed to her, did not do the necessary hygiene.”* The therapist anticipates that the client will avoid or resist exposure (Avoidant Protector, Passive Resister, Rebel mode).

(P10) *“It’s an online session. It’s a father and teenage son and the teenager has a lot of hygiene compulsions and I’m struggling to keep them together in the session, because the teenager is sitting about 50 centimeters away from his father and he does not want to touch him, because that’s part of the compulsion and the father is angry, angry that no, his compulsions have messed up the whole family and you cannot have that.”* The therapist perceives the recurring clash between the coping modes of the two (the son with OCD and his father) in which the father’s Scolding Overcontroller and the son’s Obsessive-Compulsive Overcontroller mode reinforce each other.

In the Avoidant resistance subcategory, we have the following examples:

(P5) *“I’m in the office and I have her in front of me, standing, holding her hands like a surgeon about to put on his gloves. When she enters the office she does not touch anything, doorknobs, money, absolutely nothing, and she looks carefully at the couch. There had been a client before her; she’d seen him come out and somehow she wanted to shake the couch, wipe it off; on the other hand, she did not want to put her hand on anything, grab a tissue, and..”* Therapist perceives perpetuation of avoidance in the client, indicating a resistant Avoidant Protector mode.

(P8) *“The client presents after a year and a half of absence, presents a serious situation of things and then it amazes me the difference between how the client was when he stopped collaborating until he came back (..) The client does not see how big the difference is between how I left things and how I resumed things at this point.”* The therapist perceives that, after an 18-month break in therapy, the client has relapsed and lost the self-awareness and self-control that he had previously achieved. The therapist feels frustrated by this but holds back from expressing it.

(P13) *“He′s sitting in the armchair, he’s looking down, he’s dressed in black jeans with a jacket, his jacket has not come off yet. He′s very sad, black as he came, looking down, but very anxious. (..) He does not necessarily answer me. I think he’s telling me he’s not feeling well, so he’s not saying his obsession, his concrete behavior.”* The therapist perceives the client’s avoidance of talking about his compulsions–an Avoidant Protector mode.

The following examples have been included in the Blended resistance subcategory:

(P12) “*I kept trying to explain how it works and she kept doing, no, these things I no, no, no, I cannot, no, no. She could not even listen to me. So strong was the sense of guilt, the connection between the images and the sense of guilt she had, that.. She was also quite agitated. I did not really have anyone to talk to.”* The therapist perceives an overwhelming guilt in the client which she copes with through a strong resistance to any form of exposure, indicating a Passive Resistor mode blended with Rebel mode and Avoidant Protector mode.

(P15) “*It’s a pre-session situation* [a few minutes before the beginning of the session the therapist recalls the client’s indecision from the last sessions] *where I weigh two options, one that involves continuing therapy and working with the patient on his obsessive coping and one that involves ending therapy. Somehow, in the first case, my fear is not that I will fail but that I will succeed and as a result, he will have nothing else left to lean on and the hardest moment is when I try to decide.”* The client’s indecision whether or not to change a life situation, expressed in previous sessions, puts the therapist in the position of deciding whether or not to continue therapy. The therapist did not conceptualize indecision and colluded with the client’s conflict between two modes.

The next triggers mentioned by therapists were included in the Superiority category:

(P4) *“I see Mr. X who came as usual dressed very nicely. He is a man who takes care of his aesthetics, he is well-groomed, and he has such a smile, one of those smiles of superiority that already puts me in a slightly alert state.”* The therapist perceives the client’s superiority attitude indicating a Self-aggrandizer mode.

(P6) *“He is in front of me, is slightly logorrheic, speaks from my field, describes very specifically a stressful situation, quotes a health psychology treatise so.* [T mimics an attitude of superiority] *(..) I feel constrained, I feel as if he is pushing me against a wall.”* The therapist perceives an attitude of self-confidence and superiority, a possible blended Strong and Independent Overcompensator and Self-aggrandizer mode, but also anticipates a possible attack on him if he does not comply.

(P9) *“He enters the office with very determined steps, closes the door very carefully and stays there for a few minutes (..) the client before sitting down takes the chair very calmly and looks at it, takes some lint off it (..) he tells me that I have some lint on my jacket (..) he explains how he wants to fix my laptop which is not sitting as it should and at the same time, he puts it down.”* The therapist perceives the client’s need to control everything and have things clean and in order in the office, indicating an Obsessive-Compulsive Overcontroller mode.

In the Client immobilized by conflict category were included the following triggers:

(P14) *“She had difficulty preparing for the tests, because these thoughts came up quite a lot and even blocked her ability to memorize, but she finally managed to memorize. (..) Then we discussed what she followed from the recommendations I had given her from the directions I gave when these thoughts appeared and she told me that at that point she could not apply anything else.”* The client does not apply the techniques given because she says she could not and did not trust them to work, which suggests a Helpless Surrenderer mode.

(P2) *“I see a person who is increasingly wringing their hands. I notice that he wants to tell me something, but at the same time he wants to restrain himself.”* The therapist perceives the conflict between the Healthy Adult and the Vulnerable (Shamed) Child which is in a dyad with a Shaming Parent. The conflict is followed by Helpless Surrenderer mode.

We have included the trigger from one of the situations described in the Abusive category.

(P7) *“I am given too many details, too many particulars, about the act of masturbation (..) I think deep down that it is some kind of perversion to describe these things to me and that he gets a satisfaction from describing these things in detail.”* The client gives a very detailed account of the act of masturbation—an Obsessive-Compulsive Overcontroller mode—and the therapist experiences this as sadistic and abusive.

### 3.2 Therapists’ schema modes activated in response to triggers

The therapist’s responses to the triggers were synthesized into mode processes that (1) were not displayed and (2) were displayed in relation with the client.

Therapist’s identified reactions that were not displayed in relation with the client (1) can be split into: overcompensation, surrender, avoidant, blended modes and Healthy Adult mode reactions.

In the overcompensation reactions, we included the following examples:

(P1) *“I think: Here we go again! (..) On the one hand I feel frustration. (..) Body temperature goes up, blood starts circulating a bit faster. I get physically active somehow, my breathing increases. (..) I get the idea of helping him to understand, somehow forced, more than he can carry or more than is natural, therapeutic.”* This indicates an Angry Child responding to a Demanding/Controlling Parent and a shift to Helpless Surrenderer. Then there is a switch to a Scolding Overcontroller blended with a Self-Aggrandizer mode.

(P11) *“My frustration had been building since he came in. I feel a bit of frustration at first and even a bit of insecurity. I was wondering if I was doing the right thing, if I was really using the technique correctly, if I was saying the right thing.”* This indicates frustration and helplessness (Angry Child responding to a Demanding/Controlling Parent) which the therapist copes with by ruminating/overanalyzing.

As part of the surrender reactions, we have the following examples:

(P2) The therapist declares she felt *“cold extremities, fingertips on both my hand and my toes (..) I feel a slight worry (..) I want to say, but my body cannot cope.”* The therapist is initially frightened (Scared/Abused Child with Punitive or Controlling Parent) and he (she) copes through a Helpless Surrender mode.

(P6) “*If he asks me something and I do not know. I feel constrained, I feel like he’s shoving me up against a wall and I have no room to fight back and if I do, I have no choice but to come over him. I feel like I’m losing a little bit of my patience and my relaxation that I have at the beginning of the session. I do not like evaluation situations and I keep trying to project when he’s going to stop, when I’m going to be able to get him out of this subject to take him to something else.”* The therapist feels evaluated and overpowered (Shamed Child/Abused Child with Shaming/Abusive Parent) and switches into Helpless Surrenderer mode blended with some Rumination.

(P12) “*What comes to mind is to shut up. Let things unfold. I feel discomfort in my heart area, like a kind of pressure. It’s hard for me to describe the sensation clearly a feeling of discomfort so cardiac to say. It was also a helplessness.”* The therapist is initially startled by the client’s agitation suggesting a Vulnerable Child–Demanding Parent dynamic and then gives up by activating a Helpless Surrenderer mode (no longer intervening at all until the end of the interaction).

(P15) *“I visualize myself as if the two* var*iants are one to one side, to one side and I walk around, I dangle between them as if there is a bridge between the two and I still walk like this. I feel fear, insecurity. I fear what each of them would mean. Somehow each of the choices comes with a cost and I fear what each of the costs would mean. No, it does not seem to be a perfect one. I also feel a little stuck. I mean as long as I do not go down either side, it’s OK, but if I do, it’s risky. I feel a heaviness in my chest almost like it’s hard to breathe.”* The therapist is aware of two possible courses of action in response to the client but in evaluating the pros and cons, they each seem very risky. This suggests that a Scared Child with a Punitive Parent has been triggered, followed by Worrying Overcontroller and a Helpless Surrenderer mode.

In the category of avoidant coping modes, we have the following:

(P7) *“I do not like it when I am given too many details, too many details about the act of masturbation in a man (..) I feel a bit abused, used. I feel disgusted (..) So in those moments I feel that I am being used for sexual purposes, for sexual gratification purposes. What he takes by force in those moments, he takes my attention. He wants my attention. If I were to make myself available to continue that subject, that’s probably how the whole session would go and we would practically not reach a positive result because the patient is so focused on describing these things that he’s not interested in any of my intentions, I’d practically be speaking alone.”* The therapist feels abused, used, perceives the client as totally inconsiderate of her, suggesting an Abused Child mode which she copes with through Rumination followed by an Avoidant Protector mode (the therapist changes the subject).

Blended modes were shown by more therapists:

(P3)*“How am I going to get her to touch those places she’s running away from, how am I going to get her to stop washing so much and stop spraying aghast all over the place. (..)* [Then T changes her attitude: she changes her posture, smiles with superiority, gesticulates as if in front of an audience] *I would say it’s accessing some kind of talent to convince her, to somehow try to distract her from the emotions that she has, from the fear that she has and convince her that everything she touches in that bedroom is not dangerous in any way (..) and I’m motivated to know that I’m going to get to where I want to get to, both for myself as a success of what I’m doing, that my work is not in vain, but also so that she can be functional and have a life that’s close to normal.”* The therapist feels challenged but maintains a Healthy Adult focus on the tasks of therapy. However, she then focuses on plans to convince the client that what she perceives as dangerous is not (Self-aggrandizer blended with Scolding Overcontroller mode) and giving herself a pep-talk (a kind of Self-soothing rumination).

(P4) *“I feel a bit insecure. I feel like I somehow have something to prove. I’m a little spurred on by the idea that he’s an intelligent man and that I, as a woman, have to show him that we women also have our capacity and our intelligence. I feel a sense of tension, at the same time I feel confident enough about myself and my femininity, because I generally dress neatly*, i.e.*, I am on the same key as him. (..) I feel a bit burdened that it’s my responsibility, because if I do not succeed this time either, it’s a man who will remain in pain. And I do not like people to stay in pain. And I feel responsible. I feel a difficulty at times in breathing.”* The therapists reference to being “insecure” suggests a Shamed Child (defectiveness schema). She copes by wanting to prove to him that a woman is competent and activates a Rescuer mode blended with a kind of Overcontroller mode focused on proving something to the client.

(P5) *“How helpless I felt then seeing that it was the I do not know how many sessions, I do not know how many more and she still wasn’t touching anything in the office. I also feel afraid that I will not be able to do anything with her. (..) I have a lot of questions running through my mind: What am I going to do with her if she does not get anything today? What if she refuses to do the exercises? If she takes me again with, I do not know, I do not remember, I have not monitored, I have not noticed, I do not know, I cannot, I feel powerless and sometimes I get so angry that the blood rushes to my head.”* The therapist’s Failure schema is activated (Demanding/Critical Parent with an Abused Child). At times, an Angry or Enraged Child is activated but she copes with a Helpless Surrenderer mode blended with Repetitive Unproductive Thinking (Overanalyzing, catastrophizing, flagellating).

(P8) *“It stirs up emotions of rage as far as he is concerned. (..) Here we go again! Here we go again with all the progress we have made. Yeah, not even starting over. We’re starting from the bottom. I feel a warmth in my chest and my shoulders are quite tense. I’m trying to understand what happened, how it got here, and somehow the indignation arises that the person in question, the client, does not see how big the difference is between how I left things and how I picked things up at this point. (..) I expect that if he saw what I see, he would flinch and change would come easier for him. I feel anger and I think, in my hands, stinging.”* The therapist initially feels exasperation and anger at the repetitiveness of the process. Her Enraged Child is activated and there is evidence of coping through a Self-Pity Victim mode, alternating with a Helpless Surrenderer and a Scolding Overcontroller mode.

(P9) *‘I feel some form of uncertainty about what I should, how I should approach this man who is very, very determined like this and very, very sure of himself. I’m thinking that I’ve been careless and that I have not really taken the lint off the chair and of course I’m looking around to see if there are other things that might be a disturbance to the patient in front of me. But at the same time, maybe out of carelessness, I do not want to create a discomfort and feel that this could somehow jeopardize the relationship between us. I feel nervous and very alert, I feel anxious.”* Initially he (she) gets scared and tries to please the client, suggesting a Vulnerable Child–Demanding Parent dynamic which he (she) copes with through Compliant Surrenderer mode blended with Perfectionistic Overcontroller mode.

Despite emotional activation, some of the therapists did not shift into dysfunctional schema modes and were in Healthy Adult mode throughout the exercise.

(P14) *“An emotion of frustration arises and I wonder if it really was her inability to apply or if it was actually a disbelief that those techniques could help her. (..) I wonder if there was a state of panic, if this situation did not turn into a panic attack, I think about the family context, if her parents had noticed her condition, if she had received support in this sense. I feel curious about what happened in the house at that moment. The frustration is gone.”* This suggests an Angry Child and Helpless surrenderer indicated by the initial frustration, but the therapist is able to step back and reflect on how alone the client may have been and see the client compassionately from her (his) Healthy Adult mode.

(P10) *“I think there’s a lot of pain on both sides and I do not know how to bring it out so they can see it. I feel like there’s a lot of helplessness there and it gives sadness and somehow something that goes to worry, there’s fear, but there’s also a concern about how, what do I do to still make it a session where we can connect with anger and everything they are feeling there. I feel a pressure on my shoulders. Somehow, like it’s my job to get things into a dialogue zone. I’m thinking about how to catch the bullets and translate them so that they arrive in terms of connection, of attachment, so that I can translate all the bullets so that they hurt less and catch the micro-interactions that show the need for connection, for understanding. That’s somehow what I am, like a runner from one side to the other, somehow that’s how I feel. It’s alertness, it’s not fear, it’s alertness, lots of it, alertness. I feel a tension a muscular tightness.”*.

(P13) *“I’m thinking about how I can help him. I generally react to these things in my stomach. And I’m also curious: What’s his life story? Why is he so sad”?* He (she) describes activation and feeling insecure on a more somatic level, for a short time, but manages to remain in the Healthy Adult mode.

In most cases, therapists displayed with clients (2) the Healthy Adult mode.

(P1) *“I think about the experience of others and whether I could use their experience precisely to try to show them that there are other ways of thinking. I also draw on the experience of the other participants and I challenge them to tell their point of view, to relate to their own behavior and when they felt they were in control, when they were not in control (..). I am happy to hear from the other patients present, who actually support the idea that in their case the only solution is total absence. On the one hand, I feel their alliance, but on the other hand, I empathize with this person and I do not want a conflict or a war of ideas. (..) On the one hand, I empathize with him, I do not want him to feel in any way marginalized, ostracized in his ideas, I’m somehow careful that those around him do not affect him. (..) At the same time there is also a kind of peace, a kind of acceptance, of resignation. If he does not make it, he will not make it, there’s also frustration, but it’s not maximum. It’s also an idea that okay, I’m frustrated, but I accept it.”* The therapist has good meta-awareness, is aware of internal mode processes, and reacts from Healthy Adult mode.

(P5) *“I start breathing, doing breathing exercises, regulating my anger and focusing on what happened to her during the week and discussing the topic she had to do. It’s going through my mind: ok, I’m going to adjust today to the content she comes with, I’m going to focus on the process, what’s going on, I’m going to reflect things back to her and I’m going to do my best. I feel more at ease. Further, I ask about the retrospective of the week’s homework that she had to do, which she obviously did not do, either did not have time or forgot. At first, I’m thinking OK somehow, I expected that. And then my nervousness increases. Related to how to bypass to her avoidance coping and how to better reflect it to her and how to better work with it so it does not interfere with the therapeutic process. There remains that state of non-specific agitation in my body, but I’m pretty calm, peaceful like that. We try to explore together, reflecting on what I notice, the coping modes that have been activated during the week in relation to the theme and beyond, we start to explore from there. She responds to each mirroring with yes, but, yes, but, yes, but yes, but. And I reflect that back to her as well. While doing that, I keep adjusting my inner state, somehow expecting her to do that. And it’s okay, that’s where she’s at and I’m also trying to reflect her and we are trying to reflect together on the moments and how she learned this way of coping and how it was functional for her in her past. That’s one of the things she gets and she comes up with examples from her life, telling me that’s how her mom does it all the time.”* The therapist has a good meta-awareness and manages to calm down and lead the therapeutic process in Healthy Adult mode. Even if the client’s coping is resistant, it no longer activates the therapist.

(P11) *‘I get it through my head that I need to calm down, take my time, I’ve calmed down because I know he will talk enough so I can reorganize myself. As I listen to him, I feel calmer and calmer and as I listen to him I manage to identify in his speech what I would call some talking heads, bridge heads, the bridge meaning the bridge between me and him. That is to say, to build again the path towards him. (..) When he says yes, but I am already set not to react emotionally to this remark because I am already thinking that he might be in competition with me, to prove something to me and then I reorganize according to this aspect.”* The therapist appeals to meta-awareness and manages not to act on the initial impulse so she acts out of Healthy Adult mode.

(P8) *“I’m so sorry that he does not see. (..) Further on, we talk and I show him the picture that he presents to me, and at that point I think he also starts to connect with me, really seeing the difference between how he left things and how he presents himself right now (..) I think I’m looking at his helplessness, the helplessness that he feels right now. That makes me anchor myself to look for solutions. I feel in control of the situation, I think motivated. Again, tingling in my limbs, as if I’m getting down to business.”* The therapist activates compassion and empathy for the client and this helps her not to act out the internal dynamics, but only to use the energy given by anger to seek solutions, acting out of Healthy Adult mode.

In two of the cases, the therapists acted from the coping modes initially activated.

(P7) *“I changed the subject because I see that he insists a lot (..) because I am from the therapist’s position, I cannot allow myself to make certain remarks because he would feel embarrassed, judged, criticized and then I would lose him.”* The therapist fails to manage the internal mode process and puts into action the Avoidant Protector mode, being afraid to confront the client’s coping.

(P12) *‘There was also helplessness, but I think there was also a sense of relief. Yes, it’s a sense of relief when you realize and you say to yourself, you just sit back and say, I do not know what to do in this situation and it’s totally fine.”* The therapist acts from the Helpless Surrenderer mode and does not remain in contact with the patient. He uses self-talk that might pass for Healthy Adult mode, but which is in fact a kind of Self-soothing. He does not return to the relationship and the patient does not return to therapy.

### 3.3 Strategies for accessing healthy adult mode

From the imagery exercises, we also identified several types of strategies associated with the Healthy Adult mode that therapists used to avoid displaying the initial mode processes that were activated by the client’s behavior.

#### 3.3.1 Focus on the process

In more than half of the cases, the way therapists re-entered Healthy Adult mode was through what we defined as Focussing on the process–in the sense of bringing the focus back to the therapeutic relationship in the present and observing the process from a meta perspective:

*“After that, when I said ok, I need to calm down, take my time, I calmed down because I know he will talk long enough so I can reorganize.”*

*“It crosses my mind: okay, I’m going to adapt today to the content that she comes with, I’m going to focus on the process, what’s going on, I’m going to reflect things back to her and I’m going to do the best I can.”*

*“When he says yes, but.. I’m already set to stop reacting emotionally to that remark because I’m already thinking that he might be in a competition with me, to prove something to me…”*

*“I refuse to enter the competition (..) I specified that “I do not know, I’ll do more research” and it was refreshing for me and for him; for me, in the sense that I relaxed and bodily it was much more ok and he was no longer in that posture of attacker, accuser, evaluator.”*

#### 3.3.2 Acceptance and compassion for self

Another type of strategy associated with the Healthy Adult mode that therapists turned to was acceptance and compassion for the self–acceptance of situations that the therapist cannot control, and compassion directed toward the self:

*“At the same time there is a kind of quiet, somewhat accepting, resignation. If he’s not going to make it, he’s not going to make it. (..) That brings me some peace of mind and there’s also a frustration, but it’s not maximum. It’s also an idea that okay, I’m frustrated, but I accept it.”*

*“I mean the thought crosses my mind that it might not be the right option but also a kind of reconciliation in the way that at this point, with what I know now, it’s the best thing I can do with the client (..) I feel relieved and a bit confident (..) I can breathe a bit better.”*

#### 3.3.3 Focus on patient resources

Some of the participating therapists used the strategy of accessing their own Healthy Adult mode by focusing on the client’s resources:

*“Somehow, it crosses my mind that OK, we still have something valuable, that she accepts this thing and that she notices and that she’s aware and maybe by working with this coping mode we’ll be able to get it out of the way and I’ll be able to get to her.”*

#### 3.3.4 Somatic grounding strategies

In another situation played out in the imagery exercises, what helped the therapist get back into Healthy Adult mode were strategies that focused on the body:

*“I’m starting to breathe, doing breathing exercises, regulating my anger.”*

## 4 Discussion

The aim of the present qualitative study was to explore the perspective of psychotherapists from different therapeutic orientations, from both a rational and an emotional point of view, on the difficult client–therapist dynamics in the therapeutic relationship when working with people with obsessive-compulsive disorder (OCD). Analysis of the imagery exercises revealed two broad categories of triggers for therapists, namely, Perceived resistance to change and clients perceived as having a demanding and superior attitude (Superiority) along with two other particular categories, Client immobilized by conflict and Abusive. Previous research supports the idea that dysfunctional overcompensation coping modes are particularly difficult for therapists to manage (4, 38, 39, 44). The conceptualization of obsessive-compulsive functioning from the ST perspective identifies the Obsessive-Compulsive Overcontroller mode (18, 21) and the Self-aggrandizer mode (5) as dysfunctional overcompensatory modes of coping. However, because obsessive-compulsive spectrum pathology is often comorbid with other Axis I or II pathologies (1, 2), therapists may encounter other variations of overcompensatory modes in clinical practice. The Client immobilized by conflict category included conflict between the client’s Vulnerable Child and the Healthy Adult followed by Helpless Surrenderer as a coping mode.

We divided the therapists’ reactions into mode processes that (1) were not displayed and (2) were displayed in relation with the client. For the two main categories of triggers, a specific response pattern can be observed in the mode processes that were not displayed with the client. Thus, in the case of clients who were perceived as resistant to change, who did not progress in therapy, and whose coping was perceived as rigid (Perceived Resistance to change), the therapist’s internal response involved a Vulnerable Child–Demanding Parent dynamic and was one of exasperation, the impulse to give up, usually manifested by a Helpless Surrenderer mode, along with anger, usually manifested by an Overcontroller mode, whose goal was to control, to convince the client to progress. The activation of the Vulnerable Child mode as a result of activating one’s own maladaptive schemas is not surprising (37, 45). In Pilkington’s et al.’s (37) qualitative research on ST therapists’ perceptions of the influence of their own EMSs on therapy, subjects generally mentioned feelings of inadequacy, shame, impairment, or abandonment experienced in the therapeutic relationship. Also, activating critical or demanding internal dialog corresponding to dysfunctional parent modes is not uncommon among therapists (36–38, 45). With clients perceived as demanding, as having high standards, as being difficult to please and with an attitude of superiority (Superiority), therapists reacted with an impulse to satisfy, manifested in ways such as Rescuer or Compliant Surrenderer or to demonstrate, manifesting in the impulse to overcompensate. In the case of the Client immobilized by conflict category, the Vulnerable Child–Demanding Parent dynamic activated a Helpless Surrender mode, and in the case of the Abusive category, an Abused Child–Avoidant protector mode. Previous research supports the findings, with psychotherapists frequently associating countertransference reactions with difficulty setting limits, detachment, over-functioning (37), and avoidance by changing the subject (46), over-responsivity, or helplessness (45). The majority of therapists were able to be aware of their initial reactions at the time. Thus, in the case of displayed modes in relation with the client, the behaviors enacted with clients were mostly in the Healthy Adult mode in 13 of the 15 situations. Since the exercise itself involved a high degree of vulnerability on the part of the participants in a relatively unfamiliar environment, it is possible that the desire for avoidance of negative evaluation may have been a factor. Also, the fact that the images related to a past situation that most of them analyzed afterwards, some even in supervision, because it was perceived as difficult, may have influenced the memory of that situation. In those situations in the imagery where therapists became aware during the process that a dysfunctional schema mode had been activated, they used various strategies to return to the Healthy Adult mode. We have grouped these strategies into four categories. Most often, therapists chose what we called Focusing on the process—observing the process from a meta-perspective. This is a Healthy Adult mode skill (6) and is supported by a number of other research studies that mention bringing attention back to the present moment and mindfulness as strategies therapists can use to appropriately manage EMS activation (37, 39, 45). Self-acceptance and compassion, manifested through an internal Good Parent dialog, part of the Healthy Adult mode (6), were another category of strategies used. Some therapists used body-focused strategies to reduce feelings of discomfort. These strategies included changing body positions or breathing exercises. In studies focusing on the therapeutic relationship, these types of strategies are also mentioned by other therapists (37). In fact, in literature covering ST, the Healthy Adult mode is defined as a state that involves grounding in the present, stopping dysfunctional coping behaviors, moving away from self-critical or demanding discourse, and promoting an internal dialog centered on compassion. This is consistent with our findings. The final category of strategies that therapists have used to move out of activated schema modes has also focused on the patient’s resources; specifically, the available capacities of the client’s Healthy Adult mode, which exist in some proportion in every person regardless of the severity of symptomatology (4, 5).

**Figure 1:**
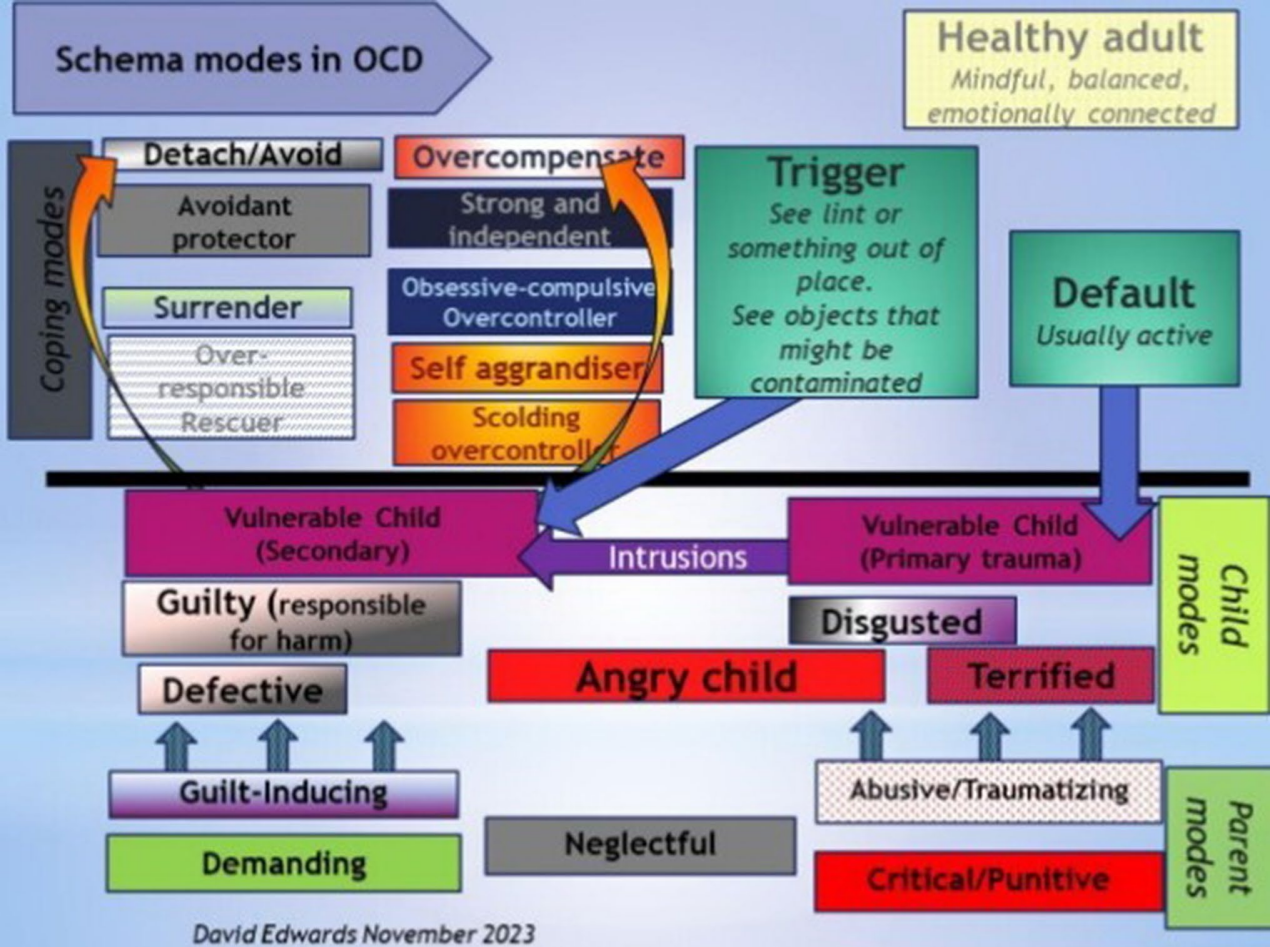
Figure drawn by D. Edwards (personal communication November 14th 2023). Used with permission.

While it was not one of our objectives, we can draw some conclusions about the client’s OCD mode system from the imagery exercises which is in line with the theories in CBT and ST literature on OCD. Thus, based on cognitive theories (21, 47) and the ST model, we can conceptualize OCD on two levels (1): a primary level related to the developmental origins of maladaptive schema (primary trauma leading to intrusive cognitions) and (2) a secondary level of schema activation in the present. From the imagery exercises, it appears that most therapists have captured and worked on the Dysfunctional Parent–Vulnerable Child dynamic of the secondary level (schema activation by a present trigger–activation of the Demanding/Guilt-inducing Parent–Vulnerable Child dynamic followed by Overcompensatory, Avoidant, or Helpless Surrender coping). The primary level, that of the origin of intrusive thoughts (Critical/Punitive Parent–Vulnerable child–Angry child dynamic) was not addressed but is captured in Figure 1 (D. Edwards, personal communication, November 14, 2023.

In conclusion, the schema modes of OCD sufferers or their family members who are involved in the therapy may activate in the therapists their own schema modes. There is a risk that the client–therapist dyad will develop what is known as a “chemistry of modes” (4). This can have a negative impact on the entire therapeutic process. Therapists use a variety of strategies to help them return to Healthy Adult mode in order to mitigate this risk. Future research would be useful and necessary to better understand the client–therapist relationship in working with people with OCD, for example, focusing on capturing clients’ perspectives of interactions with therapists at key moments or analyzing interaction sequences captured in real time.

## 5 Limitations

One of the important limitations of the present study is related to the representativeness of the sample. The therapists who participated in the research, as well as their clients, are part of the same small geographical area so that different cultural aspects may bias the results. In our research, we have found that, in addition to the OCD-specific coping modes, namely, the Obsessive-Compulsive Overcontroller and the Avoidant modes, other schema modes also play a relevant role in the therapeutic relationship with these clients. Although the schema modes are universal, the frequency with which they are encountered is likely to be different in different cultures. In Romanian society, which has been marked by decades of dictatorship, certain types of defensive reactions, such as victimization, helpless surrender, or shifting of responsibility, with a tendency to overestimate the importance of social image and fear of evaluation, are very common and applicable to both clients and therapists. Furthermore, the subjects represent only a small fraction of the therapeutic orientations in clinical practice. Future research on how OCD clients from different cultures interact with their therapists will therefore be able to differentiate to what extent identified patterns can be generalized. Another important limitation stems from the technique used to capture the dynamic between the therapist and the client. With the imagery exercise, we had access only to the therapists’ perspective on the situations described, as they remained fixed in memory (41, 42). The schema modes of OCD sufferers were described as they were perceived by the therapist and not from direct observation. At the same time, most of the participating therapists were unfamiliar with imagery exercises. Given that the exercise itself involved a high degree of vulnerability on the part of the participants in a relatively unfamiliar environment, it is possible that the desire not to be judged negatively or to maintain a certain professional image may have influenced responses, and this should be considered a limitation of the study. Also, the fact that the imagery referred to a past situation that most of them had analyzed afterwards, some of them even in supervision, precisely because it was perceived as a difficult situation, may have influenced the memory of this situation, and this aspect is also a limitation of our research.

### 5.1 Clinical recommendations

We have several recommendations with respect To training and supervision for therapists working with OCD:

- Focus on the case conceptualization of OCD cases. This would help with normalizing the challenging coping modes these clients bring to therapy.
- Provide strategies for empathic confrontation.
- Educate therapists about treatment-resistant cases and the fact that there are many cases of OCD that do not respond to outpatient treatment alone.
- Help therapists recognize their own triggering and triggered mode patterns and strengthening their capacity to stay grounded and cultivate meta-awareness.
- Use the kind of imagery exercise used here to heighten therapists’ awareness of the mode cycles between them and the client that can emerge when working with OCD and how to navigate them.
- Guide therapists in recognizing alliance ruptures and acting to repair them as a therapeutic priority.

### 5.2 Treatment strategies

The schema therapy approach offers a range of strategies for dealing with situations where the client is not ready to engage in behavioral exposure. These include chair work with the coping modes, mode interview, and imagery rescripting with future-self. To support clinicians working with complex cases of OCD and who might be confronted with situations similar to those of the study participants, we offer a number of suggestions for overcoming resistance.

Specific recommendations for treatment strategies:

- Conceptualize both the current problem and the developmental origins of maladaptive schemas/modes with the client.
- Starting from the emotions activated by intrusive cognitions, access the primary vulnerability through affective bridge using the Imagery rescripting (ImR).
- Elicit the developmental history of Coping child modes (6) (building a behavioral bridge–e.g., when you first started checking) using empathic confrontation when they become active in the therapeutic process, either through chair work or mode interview. Using empathic confrontation, the client is invited to become aware of their coping child, which is the origin of the present coping mode, helping to build a more compassionate stance for the mode and bypassing it more easily.
- Both the therapist’s empathy and respect for the client and the client’s self-esteem can be enhanced and the therapeutic relationship can be opened or repaired by identifying the primary role and purpose of coping modes.
- Create mode shift chains with the client as part of the conceptualization and normalization of the OCD process (Trigger–Punitive Parent–Vulnerable Child–Coping Mode–Demanding Parent–Vulnerable Child–Coping Mode) (21).

These strategies, when implemented with careful consideration, can help to overcome therapeutic impasses. They also contribute to the strengthening of the therapeutic relationship and to limited reparenting because they involve empathy, guidance, and a focus on the client’s needs.

## Data availability statement

The original contributions presented in the study are included in the article/supplementary material, further inquiries can be directed to the corresponding author.

## Ethics statement

The studies involving humans were approved by Research Ethics Committee of the Faculty of Psychology and Educational Sciences of Alexandru Ioan Cuza University. The studies were conducted in accordance with the local legislation and institutional requirements. The participants provided their written informed consent to participate in this study.

## Author contributions

SS, MS, SB, CS, and OG: data curation, data analysis, and writing–review and editing. SS conducting interviews, conceptualization, writing–original draft, and data analysis. SS and CS designed the research. All authors contributed to the article and approved the submitted version.
